# Context-Aware Fusion of RGB and Thermal Imagery for Traffic Monitoring

**DOI:** 10.3390/s16111947

**Published:** 2016-11-18

**Authors:** Thiemo Alldieck, Chris H. Bahnsen, Thomas B. Moeslund

**Affiliations:** Visual Analysis of People Lab, Aalborg University, 9000 Aalborg, Denmark; tbm@create.aau.dk (T.B.M.)

**Keywords:** context-aware fusion, traffic surveillance, segmentation

## Abstract

In order to enable a robust 24-h monitoring of traffic under changing environmental conditions, it is beneficial to observe the traffic scene using several sensors, preferably from different modalities. To fully benefit from multi-modal sensor output, however, one must fuse the data. This paper introduces a new approach for fusing color RGB and thermal video streams by using not only the information from the videos themselves, but also the available contextual information of a scene. The contextual information is used to judge the quality of a particular modality and guides the fusion of two parallel segmentation pipelines of the RGB and thermal video streams. The potential of the proposed context-aware fusion is demonstrated by extensive tests of quantitative and qualitative characteristics on existing and novel video datasets and benchmarked against competing approaches to multi-modal fusion.

## 1. Introduction

In order to increase road safety or address the problems of road congestion, one must obtain a thorough understanding of road user behavior. Such an understanding may be derived from detailed, accurate information of the traffic. Video surveillance offers a rich view of a traffic scene and enables 24-h monitoring at a fairly low cost [1]. Manual observation of the traffic scene is a tedious and time-consuming task, however, and automated techniques are thus desired. Computer vision techniques enable the automatic extraction of relevant information from the surveillance video, such as the position and speed of the traffic and the classification of the road user types [2].

The use of cameras for monitoring purposes, however, introduces a significant drawback. As the functional principle of a camera builds on the visual range of light, the quality of the data is highly dependent on environmental conditions, such as rain, fog and the day and night cycle. As a result, many applications work only during the daytime in decent weather conditions, and a persistent monitoring of the scene is often desired. Although custom methods have been proposed for specialized scenarios [3,4,5,6,7,8], a standard method for different purposes and under arbitrary conditions is yet to be presented.

To overcome this problem, both sensors and algorithms must be designed for long-term persistence under varying, real-world conditions. On the sensor side, one solution is to supplement the traditional visible light camera with other sensor types. Such multi-sensor systems are more persistent to changes in the environment; if the output of one sensor is impaired due to sub-optimal conditions, other sensor types are not necessarily affected.

**Figure 1:**
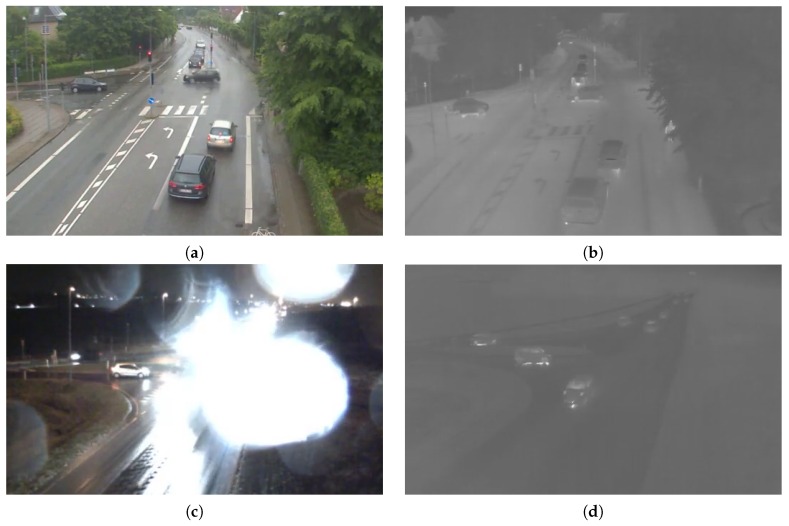
RGB and thermal images of two scenes. In the top scene, the RGB image (**a**) provides a detailed view of the road users. The thermal image (**b**) shows fewer details, but gives a better view of the pedestrian behind the tree on the pavement. In the bottom scene, the headlights of the approaching vehicles blurs parts of the RGB image (**c**) and introduces glare by the raindrops on the lens. Fortunately, the corresponding thermal image (**d**) is unaffected by the headlights.

Consequently, a special interest in thermal infrared cameras has recently developed. Thermal cameras cannot capture visible light, but only pick up the infrared radiation emitted by objects [9]. The infrared radiation depends on the temperature of the object, thus making the imaging system independent of illumination and less dependent on visual obstructions caused by, e.g., fog or rain. As seen in Figure 1, the downside is that thermal images are less detailed and provide an unfamiliar visual impression to a human observer. When combined, traditional visible light (RGB) cameras and thermal cameras enables 24-h surveillance under arbitrary lighting conditions and improve the observability under challenging environmental conditions.

In order to utilize the information from the various sensors, one should fuse the information at some point in the data processing chain. However, how is the data fusion actually performed? When fusing the data streams, how should the different streams be weighted against each other? In the ideal case, the weights are dependent on the information quality of the data stream of a particular sensor, e.g., how objects of interest are distinguished from other parts of a scene. The information quality of a data stream is dependent on the sensor attributes, the object nature, scene geometry and environmental conditions, but also on the purpose and nature of the subsequent analysis of the data.

In this paper, we present a novel method for the context-based fusion of video from thermal and RGB video streams. The context-based fusion is integrated with the segmentation of the scene, which is the first and crucial step of bottom-up processing pipelines [2] commonly used in real-time surveillance systems. We integrate the contextual information of the scene to assess the quality of the video data, which we use to fuse the output of two parallel segmentation pipelines.

The methodology of image fusion and related work is discussed in Section 2. In Section 3, we deduce context-based quality parameters based on environmental conditions and the appearance of the video data. These parameters are used to design a context-adaptive fusion pipeline, which is described in Section 4. This pipeline is exemplified using an image segmentation algorithm in Section 5 to create a fused, segmented image, which is common to both the thermal and RGB video streams. Subsequently, we present extensions for the application of traffic monitoring in Section 6. In Section 7, we evaluate the context-based fusion on our own and two commonly-used datasets against competing approaches to image fusion. Finally, our conclusions are presented in Section 8.

## 2. Related Work

Different sensors have advantages and disadvantages in terms of further processing. To overcome the individual downsides of different sensors, multimodal systems have been developed. These systems use information from multiple sensors and information sources to combine and enrich the available data. The potential of these methods, especially for traffic surveillance, has been emphasized by Buch et al. [2]. In this section, different fusion approaches will be presented and discussed. The main focus will thus be on the fusion of video data from thermal and RGB cameras.

Fusion approaches are generally divided into three levels: pixel-level fusion, feature-level fusion and decision-level fusion, depending on the stage at which the fusion takes place [10].

Decision-level fusion combines the output from two or more parallel processing pipelines. The results are merged by Boolean operators or the weighted average. Serrano et al. [11] perform parallel segmenting of thermal and RGB data and select the representative output on the basis of confidence heuristics.

Feature-level fusion takes place one step earlier in the processing pipeline. Features from all input images are extracted individually and then fused into a joint feature space. Kwon et al. [12] used this technique for automatic target recognition.

Pixel-level fusion is the most common approach. In this type of fusion, the input images are merged into one. Details that might not be present in one image are hereby added by the other modality. Common examples are structures occluded through dark shadows or smoke in RGB images that are revealed with the help of a thermal image. Pixel-level fusion requires all input images to be spatially and temporally aligned. This alignment, also called registration, is a challenge. Automatic image registration approaches often fail as there is no correlation between the intensity values of the modalities [13]. A common approach is to manually select corresponding points in both modalities and compute a homography. However, special-case automatic methods exist; these use features that are most likely present in both modalities, e.g., contours [14], Harris corners [15] or Hough lines [16].

Shah et al. [17] perform the fusion after different wavelet transforms of the images. This allows a fusion rule based on frequencies rather than pixels. The approach preserves the details while still reducing artifacts. Chen and Leung followed a statistical approach in [18] by using an expectation-maximization algorithm.

Lallier and Farooq [19] perform the fusion through adaptive weight averaging. The weight per pixel is hereby defined by the number of equations that express the interest in the specific pixel. In the context of this work, these are the degree to which an object is warmer or colder in the thermal domain, the occurrence of contrast differences and large spatial and temporal intensity variations in the visual domain.

Instead of fusing the images to a new image, which can be represented in RGB, other methods simply combine the inputs into a new format. St-Laurent et al. [20] adapt a Gaussian Mixture Model (GMM) algorithm for extracting moving objects to work with “Red-Green-Blue-Thermal” (RGBT) videos. In this way, important information is automatically revealed by the object extraction algorithm.

## 3. Context-Based Image Quality Parameters

In this work, we use a pixel-level fusion approach. However, unlike usual pixel-level approaches, the RGB and thermal images are not fused immediately. Instead, we use the soft segmentation results from individual processing of the thermal and RGB video streams. The quality of the video streams is used to fuse the soft segmentation results and, thus, forms a context-aware, quality-based fusion.

In the following, we discuss the conditions that effect the image quality for surveillance scenarios and how those conditions may be predicted by data from different sources. The aim is to construct context-sensitive indicators, $q_{\mathrm{RGB}}$ and $q_{\mathrm{thermal}}$, that express the usefulness of each modality.

When assessing the relative qualities of the thermal and RGB images, we distinguish between predictable and unpredictable conditions. The predictable conditions are considered “static” under short time spans, but may change gradually over several hours, such as the position of the sun or the general weather conditions. The unpredictable conditions cannot be measured beforehand and may change rapidly in a few seconds, for example when a cloud temporarily blocks the Sun. In the following, we start by discussing the predictable conditions in terms of the thermal and RGB images, which is followed by a discussion of the unpredictable conditions.

### 3.1. Predictable Thermal Image Quality Characteristics

Thermal cameras measure the infrared radiation emitted by all objects. The energy of the radiation mainly depends on the object temperature. A constant factor, referred to as emissivity, scales the radiation for different materials [9]. If the emissivity is known, the temperature of objects obtained in thermal images can be calculated using the Stefan–Boltzmann law [21]. However, Automatic Gain Control (AGC) often forms part of many of the thermal cameras that are built for surveillance, and this implies that the exact relation between radiation energy and intensity values is often unknown.

Objects consisting of different materials have different intensity values in a thermal image, even if they have almost the same temperature [9]. Typical scenes consist of several different materials, and we therefore expect a certain amount of information in the thermal image; also for scenes without foreground objects. If no objects can be distinguished, the information content is low. Consequently, the image entropy can be used as a quality indicator for thermal images. The entropy, *H*, is defined as:

(1) $$H=-\sum_{i=0}^{255}p\left(I_{i}\right)\frac{\log(p\left(I_{i}\right))}{\log(2)}$$

where $p(I_{i})$ is the percentage of pixels with intensity *i* in the thermal image *I*.

**Figure 2:**
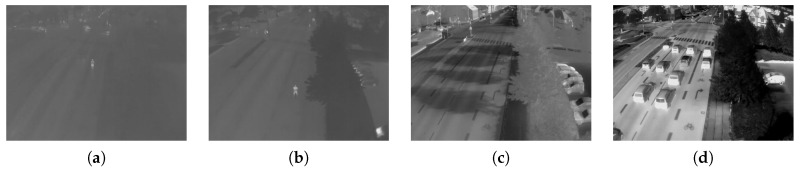
Thermal images of the same scene with different entropy values. (**a**) H=4.23; (**b**) H=5.04; (**c**) H=6.65; (**d**) H=7.67.

Figure 2 shows a side-by-side comparison of the same location at different times. The right images appear much more detailed and, therefore, of higher quality. The corresponding entropy values correlate with this impression.

Experiments have been conducted and have shown that a linear function enforces a too strong down-rating of low entropy values. Thus, a sigmoid function is found to be a better approximation of the mapping function between the entropy and quality of the thermal image. Thus, the entropy quality parameter is defined as:

(2) $$q_{\mathrm{entropy}}=\frac{1}{1+e^{(3.5-H)\cdot 2}}$$

**Figure 3:**
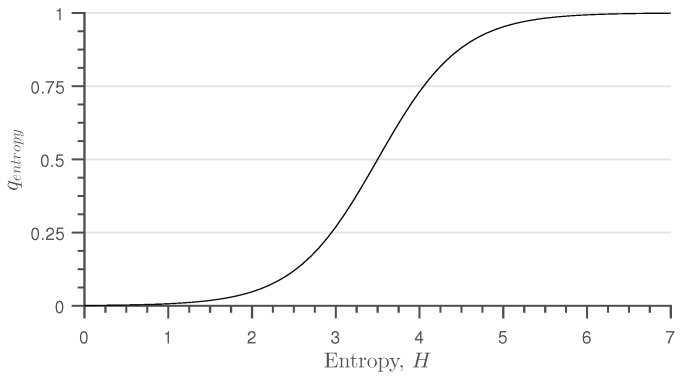
Shape of the $q_{\mathrm{entropy}}$ quality function relative to the entropy of the thermal image.

The shape of $q_{\mathrm{entropy}}$ is shown in Figure 3.

### 3.2. Predictable RGB Image Quality Characteristics

The predictable image quality of an RGB image is closely correlated with the amount of light in the scene. When working with outdoor scenes, the amount of light in the scene is strongly dependent on the available sunlight. The more sunlight, the higher the image quality. However, in full sunlight, shadows will appear, which might be the cause of false positives when segmenting the image. The state of the weather in a scene is pivotal when estimating the general observability of the scene. Phenomena such as mist and fog reduce the visibility. Rain and snowfall introduce spatio-temporal streaks in the image, which further impedes the view.

In the following, we will discuss the effect of these phenomena on the RGB image quality.

#### 3.2.1. Illumination

**Figure 4:**
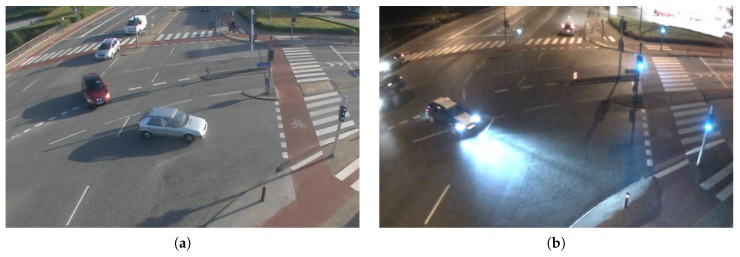
RGB images with common challenging conditions. (**a**) Shadows; (**b**) Reflections and halos.

Figure 4 shows the same scene in the afternoon and at dusk. While a human being can easily label the cars in the scene, segmentation algorithms would be highly disturbed by the large shadows and reflections. Although several shadow suppression algorithms exist nowadays [22], shadows still disturb the detection process. The handling of reflections, as imposed by moisture and shiny surfaces, is still an unsolved problem. In conclusion, both images presented in Figure 4 should be rated as low quality, although the reasons for the low quality are different and so may the quality rating be.

**Figure 5:**
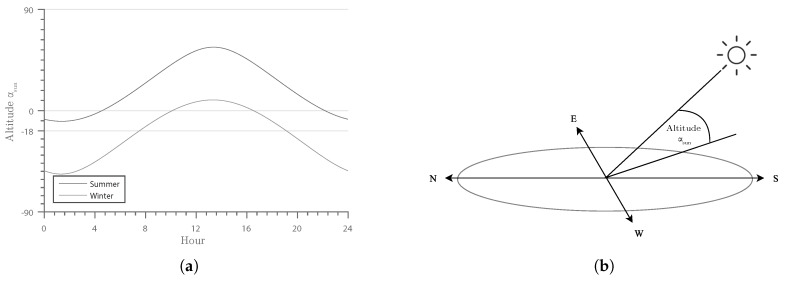
Solar altitude over a day in the summer and winter (**a**); the solar altitude is defined by the angle between the ground plane and the Sun’s position vector (**b**).

**Figure 6:**
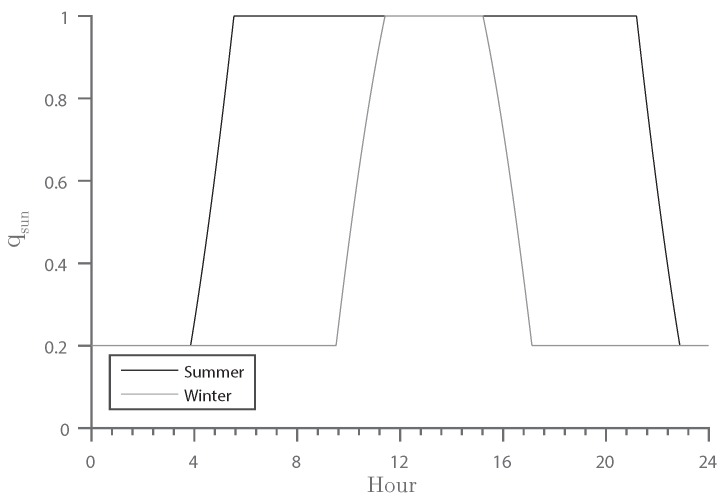
Development of the $q_{\mathrm{sun}}$ quality indicator over a winter and summer day. The corresponding altitude of the Sun is shown in Figure 5a.

Consequently, images with low light conditions, such as twilight and night, should be rated as low quality. The elevation angle of the Sun, as illustrated in Figure 5, can be used as an input parameter. The solar elevation angle, $\alpha_{\mathrm{sun}}$, is defined as the angle between the ground plane and the Sun’s position vector; see Figure 5b. It is dependent on the longitude and latitude of the scene, as well as the date and time of the recording [23]. The Sun is visible for angles ≥$0^{\circ }$. In practice, however, noticeable illumination is not present before $-6^{\circ }$, known as civil twilight [24]. Additionally, as soon as the Sun is visible, the illumination condition is not perfect. Therefore, in this work, we require the altitude of the Sun to increment an additional $6^{\circ }$ before we define good illumination and, thus, set $q_{\mathrm{sun}}=1$. If the altitude of the Sun is below $-6^{\circ }$, the Sun does not contribute to the light in the scene, and we set $q_{\mathrm{sun}}=0$. However, there might be other light sources that contribute to the illumination of the scene, for example street lights. Thus, we define a non-zero minimum quality parameter, $q_{\mathrm{smin}}$. This leads to the following formula:

(3) $$q_{\mathrm{sun}}=\left\{\begin{array}{cc} 1.0 & \mathrm{if}\;\alpha_{\mathrm{sun}}\geq 6^{\circ }\\ \text{max}\left(\frac{\alpha_{\mathrm{sun}}+6^{\circ }}{12^{\circ }},q_{\mathrm{smin}}\right) & \mathrm{if}\;-6^{\circ }\leq \alpha_{\mathrm{sun}}<6^{\circ }\\ q_{\mathrm{smin}} & \mathrm{if}\;\alpha_{\mathrm{sun}}<-6^{\circ } \end{array}\right.$$

with the solar elevation angle $\alpha_{\mathrm{sun}}$ and a minimum quality parameter $q_{\mathrm{smin}}$, which is set according to the amount of artificial light available in the scene. The resulting function is displayed in Figure 6 with $q_{\mathrm{smin}}=0.2$.

#### 3.2.2. Shadows

Two external factors specify the occurrence of the shadows. First of all, shadows may appear only on sunny days. Sunny days may be detected by integrating a weather station next to the setup or by accessing a weather database. The length of these shadows is determined by the Sun’s position. Therefore, both weather data and the solar elevation angle must be considered to present a model showing to what extent cast shadows might be present in the scene. The length of shadows can be calculated through:

(4) L=h/tan(α)

with *h* being the object height. With unit object height, Equation (5) can serve as a quality function, where *ψ* is a scaling factor, $q_{\mathrm{weather}}$ is the weather quality indicator defined in Section 3.2.3 and $q_{\mathrm{shmin}}$ is the minimum required quality.

(5) $$q_{\mathrm{shadows}}=\left\{\begin{array}{cc} \text{max}(1-\psi L,q_{\mathrm{shmin}}) & \mathrm{if}\;\alpha_{\mathrm{sun}}>0\wedge q_{\mathrm{weather}}=1\\ 1.0 & \mathrm{otherwise} \end{array}\right.$$

**Figure 7:**
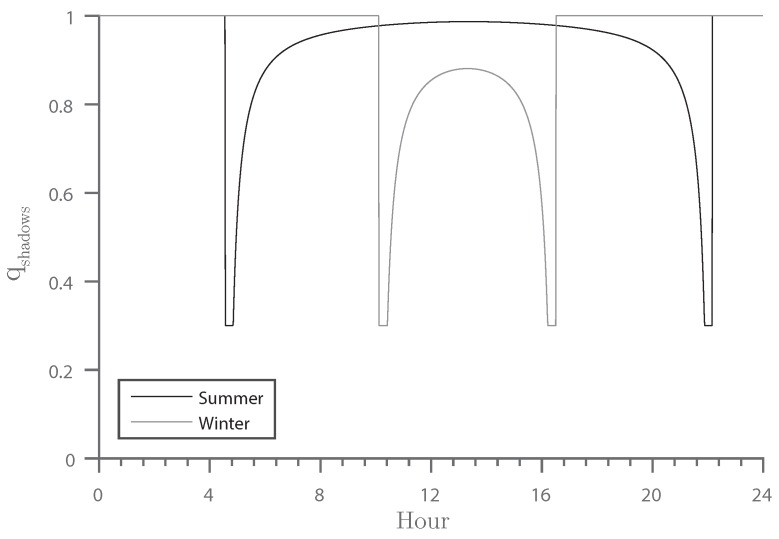
Development of the $q_{\mathrm{shadows}}$ quality indicator over a day during summer and winter. The corresponding altitude of the Sun is shown in Figure 5a.

The resulting function is plotted in Figure 7 with $q_{\mathrm{shmin}}=0.3$ and ψ=50.

#### 3.2.3. Weather Conditions

Different weather conditions may harm segmentation algorithms through various phenomena, such as mist, fog, rain and snow. The long-term effects of rain are visible as reflections in puddles and moisture on the road. A quantitative rating, however, is not so easily derived. For this work, we have grouped weather conditions obtained from [25] into five broad categories, as seen in Table 1. A clear sky is defined as optimal conditions with a quality rating of one. Clouds and light mist reduce the amount of light available in the scene and are as such assigned a lower quality rating of 0.8. The occurrence of rain and snow induces spatio-temporal noise and reduces the visibility of the scene. We distinguish between light and heavy rain and all other types of precipitation. The spatio-temporal effects of light rain are barely visible, whereas raindrops may be visible during heavy rain, snow and hail [26]. Fog and haze do not occur as spatio-temporal effects, but greatly reduce the visibility and are thus grouped with heavy rain and snow.

### 3.3. Unpredictable Image Quality Characteristics

We define unpredictable conditions as rapidly changing, dynamic conditions that may not be predicted by the sensors or the available contextual knowledge. In the RGB image, this includes rapidly changing illumination, for instance caused by clouds that temporarily blocks the Sun. In the thermal image, the most prominent, dynamic change is caused by the auto-gain mechanism of the thermal camera. The auto-gain automatically maximizes the contrast of the thermal image by adjusting the gain of the camera, which means that the appearance of a scene may change suddenly when cold or warm objects enter the scene.

Because the rapidly-changing conditions may not be predicted beforehand, we will rate them by their effect on the subsequent image segmentation process. Typically, most segmentation algorithms will respond to rapidly-changing conditions with abrupt changes in the ratio of Foreground (FG) and Background (BG) pixels. Over time, the segmentation algorithm will incorporate the changes, and the ratio of FG and BG pixels stabilizes.

We can incorporate this characteristic in a quality indicator, such that rapid changes in the FG/BG ratio are penalized. This indicator, $q_{\mathrm{fg}}$, is defined in Equation (6), where *τ* defines the average foreground ratio and *γ* is a weight controlling the foreground deviation:

(6) $$q_{\mathrm{fg}}=\text{max}\left(1-\gamma \left(r_{\mathrm{fg}}-\tau \right),0\right)$$

where the current foreground ratio, $r_{\mathrm{fg}}$, is defined as:

(7) $$r_{\mathrm{fg}}=\frac{1}{XY}\sum_{x=1}^{X}\sum_{y=1}^{Y}𝟙$$

where 𝟙 denotes an indicator function that returns one if the image at position (x,y) is foreground, otherwise zero, and (X,Y) are the image dimensions.

The $q_{\mathrm{fg}}$ indicator is computed separately for the RGB and thermal image streams.

### 3.4. Combined Quality Characteristics

At this stage, we have developed several indicators for the image quality of both modalities, which should be combined into one quality indicator for each modality. We start by combining the indicators that correspond to the static predictable conditions. In the thermal domain, this is easy, as there is only one indicator, $q_{\mathrm{entropy}}$, to consider. In the RGB domain, the quality indicators are closely interrelated. However, the exact nature of these relations is unknown, and a study of this is beyond the scope of this work. Therefore, we here assume decorrelation and hence combine the indicators by multiplication:

(8) $$q_{{\mathrm{static}}_{\mathrm{RGB}}}=q_{\mathrm{sun}}\cdot q_{\mathrm{shadows}}\cdot q_{\mathrm{weather}}$$

(9) $$q_{{\mathrm{static}}_{\mathrm{Thermal}}}=q_{\mathrm{entropy}}$$

The predictable and unpredictable quality indicators are combined for each modality by taking the minimum value:

(10) $$q_{\mathrm{RGB}}=\min\left(q_{{\mathrm{fg}}_{\mathrm{RGB}}},q_{{\mathrm{static}}_{\mathrm{RGB}}}\right)$$

(11) $$q_{\mathrm{Thermal}}=\min\left(q_{{\mathrm{fg}}_{\mathrm{Thermal}}},q_{{\mathrm{static}}_{\mathrm{Thermal}}}\right)$$

To prevent artifacts, the quality indicators are gradually updated:

(12) $$q_{t}=\left\{\begin{array}{cc} q_{t} & \mathrm{if}q_{t}\leq q_{t-1}\\ \alpha q_{t}+\left(1-\alpha \right)q_{t-1} & \mathrm{otherwise} \end{array}\right.$$

where *α* is the update rate of the segmentation model. The calculation is performed independently in the RGB and thermal domain.

## 4. Context-Based Fusion

**Figure 8:**
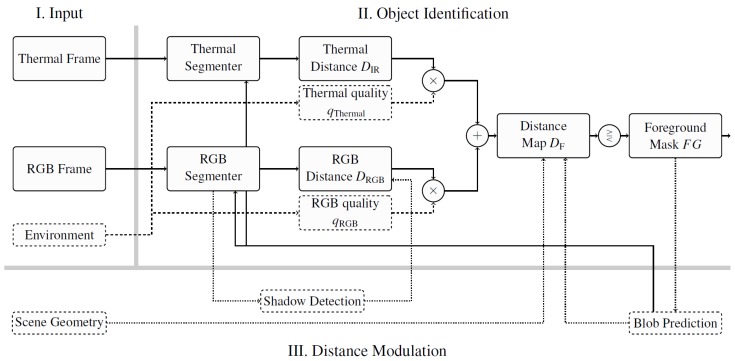
System design overview: The three main phases of the fusion algorithm are illustrated. Two registered input streams are processed by two parallel segmentation algorithms. The soft segmentation results from these algorithms, denoted as distance maps, are fused by using the quality indicators of each stream. Distance modulation functions may improve the algorithm for the purpose of traffic monitoring by using constraints derived from scene geometry, shadow detection and object (blob) detection; see Section 6.

The following section presents a new approach to fusing the image streams by integrating the quality indicators into a segmentation pipeline. As opposed to other works, we do not fuse the input data directly. Rather, we have used the intermediary results of two parallel segmentation algorithms. The results are weighted in accordance with the quality indicators described before to ensure that the system is context-aware. Figure 8 illustrates the basic principle of this work. The core contribution is illustrated in Part II, object identification, of Figure 8. The images of the thermal camera are registered into the coordinate system of the RGB image by using a planar homography [27] such that positions on the road plane in the thermal image correspond to the same positions in the RGB image. The registered images are fed into two parallel segmentation algorithms from which we get the intermediate, soft segmentation results that represent, for each pixel, the degree of belief that the pixel is considered to be in the foreground. In this work, we denote this as the distance maps. The fusion of these maps is discussed in the following. The details of Part III, distance modulation, of Figure 8 are explained in Section 6.

We normalize the quality indicators $q_{\mathrm{RGB}}$ and $q_{\mathrm{Thermal}}$ to add up to one and use the normalized values as weights for the adaptive fusion of the distance maps. The weights are calculated as follows:

(13) $$\begin{array}{ccc} w_{\mathrm{RGB}}=\frac{q_{\mathrm{RGB}}}{q_{\mathrm{RGB}}+q_{\mathrm{Thermal}}} & \; & w_{\mathrm{Thermal}}=\frac{q_{\mathrm{Thermal}}}{q_{\mathrm{RGB}}+q_{\mathrm{Thermal}}} \end{array}$$

The distance map of each modality is multiplied by its corresponding weight, and the results are summed to create a unified, fused distance map:

(14) $$D_{F}=w_{\mathrm{RGB}}D_{\mathrm{RGB}}+w_{\mathrm{Thermal}}D_{\mathrm{Thermal}}$$

At this stage, small inaccuracies in the spatial and temporal registration can be compensated. A simple mean filter applied on the fused distance map dissolves the pixel grid and therefore fuses information from neighboring pixels.

The final step in the segmentation is the decision as to whether a pixel is defined as foreground or background. We threshold the fused distance map on a per-pixel level:

(15) $$\mathrm{FG}=\left\{\begin{array}{cc} 1 & \mathrm{if}D_{F}\geq T\\ 0 & \mathrm{otherwise} \end{array}\right.$$

where *T* is the segmentation threshold, usually set to one.

## 5. Segmentation Algorithm

In the framework presented in the previous section, we fused the intermediate output of two segmentation algorithms. Any image segmentation algorithm may be used, as long as it generates a soft-decision pixel map that may be used as the distance map of Figure 8. In the rest of this paper, we apply a particular segmentation method in order to be able to quantify the benefits of the proposed fusion strategy. We use the classic Gaussian Mixture Model (GMM) [28] to exemplify our context-fusion framework.

The GMM is widely used within the domain of traffic surveillance [2] and represents a well-known platform to showcase the context-based fusion. A brief introduction to the GMM is given in the following.

During the calculation of the background distance based on the GMM, each pixel is tested against each component of the GMM’s background model. The Mahalanobis distance of the sample value from the background model is hereby the determining factor for acceptance. A pixel *x* at time *t* is defined to match the background component if it falls within *λ* standard deviations:

(16) $$M_{i,t}=\left(\frac{|x_{t}-\mu_{i,t-1}|}{\lambda \sigma_{i,t-1}}<1\right)$$

where $M_{i,t}$ is the *i*-th background model at time t+1, $\mu_{i,t-1}$ and $\sigma_{i,t-1}$ is the mean value and standard deviation of $M_{i,t-1}$, respectively.

The mean and standard deviation of the background models are constantly updated as follows:

(17) $$\mu_{i,t}=\left(1-\beta \right)\mu_{i,t-1}+\beta x_{t}$$

(18) $$\sigma_{i,t}^{2}=\left(1-\beta \right)\sigma_{i,t-1}^{2}+\beta {\left(x_{t}-\mu_{i,t-1}\right)}^{2}$$

where *β* is defined as:

(19) $$\beta =\alpha N(x_{t},\mu_{i,t-1},\sigma_{i,t-1}^{2})$$

and *α* is a constant update rate.

The acceptance distance of the sample as the foreground in Equation (16) is normalized by the specific variance $\sigma_{i,t}$ and the threshold value *λ*. Large distance values indicate a high probability of the pixel being in the foreground, whilst small values show high conformity with the component. With this in mind, an approximation of the general conformity of a pixel in the model can be expressed by computing the distance value, $D_{t}$:

(20) $$D_{t}\approx \left\{\begin{array}{cc} d_{0,t} & \mathrm{if}\;M_{0,t}\\ d_{1,t} & \mathrm{if}\;M_{1,t}\\ & \ldots \\ d_{b,t} & \mathrm{if}\;M_{b,t}\\ \text{min}(d_{0,t},d_{1,t},\cdots ,d_{b,t}) & \mathrm{otherwise} \end{array}\right.$$

with:

(21) $$d_{i,t}=\frac{|x_{t}-\mu_{i,t-1}|}{\lambda \sigma_{i,t-1}}$$

where *b* denotes the total number of background models.

**Figure 9:**
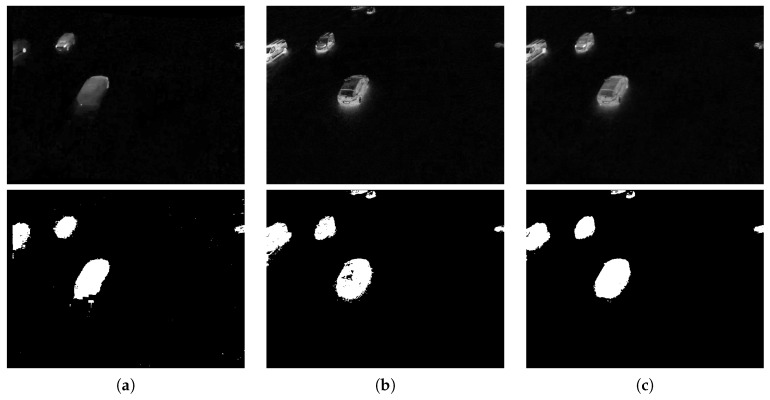
Distance maps of the different modalities and results after thresholding. The intensity of a pixel corresponds to the distance to the background model of each parallel segmentation algorithm. Bright pixels indicate a high probability for foreground objects. (**a**) Thermal; (**b**) RGB; (**c**) fused.

If a match $M_{i,t}$ is found, the corresponding value of $d_{i,t}$ is used to express the distance. Otherwise, the distance to the closest component is used. The resulting values of all pixels form a map expressing the deviation of image regions from the background, and this is fed into the context-based fusion framework as the distance map. Figure 9 displays the distance maps, their fusion and the effect on the resulting mask.

## 6. Application to Traffic Monitoring

The preceding sections described the main contribution of this work. In the following, we will present specific extensions for traffic surveillance to show the modularity of the proposed algorithm. In Figure 8, these extensions are categorized as III, distance modulation.

### 6.1. Shadow Detection

A common extension of background modeling techniques is shadow detection. Shadows of intruding objects do not match the background model; they appear as darker formerly-illuminated areas and are, therefore, defined as foreground. Depending on the purpose of the system, the labeling of shadow areas as foreground is a false positive error. In most surveillance scenarios, only the objects (not their shadow) are of interest [22].

Prati et al. [22] distinguish between deterministic approaches to shadow detection, which use an “on/off decision process”, and statistical approaches, which “use probabilistic functions to describe the class membership”. However, both methods can fail and lead to false negatives, just as false positives may also occur. If the main task is to identify all foreground objects, as in the case of traffic surveillance, especially false positives may harm the results. Whole objects may be classified as shadows. To address this issue, shadow areas have been pruned rather than completely removed in this work.

State-of-the-art methods perform a labeling function in the resulting foreground mask. Instead of making this hard decision, the distance related to the areas marked as shadows is scaled down. In this work, a fixed scaling method has been used. A scaling based on the shadow certainty may be a possible extension. As the background distance correlates with the certainty of a pixel being in the foreground, the downscaling may be considered as bringing uncertainty to the decision. Consequently, the decision of whether a pixel is defined as shadow is only made indirectly when deciding whether the pixel is categorized as either foreground or background.

The subsequent fusion of the modalities is the important step for this method to work. Objects that have also been found in the thermal image are most likely found anyway, and shadows are voted further down as they are not present in the thermal domain. Especially small areas of false positives can be recovered as being a foreground object using this technique. The mean filter subsequent after the fusion helps the process of removing outliers. Additionally, the quality indicators allow prediction of scenes with shadows. Therefore, the process can be triggered to be context aware.

### 6.2. Blob Prediction

A successful segmentation algorithm for traffic surveillance must handle the different speeds of the traffic, which implies that all objects must be handled as foreground even when staying in the scene for a longer time. For this purpose, the blob prediction method proposed by Yao and Ling [29] has been integrated in this work. The position of foreground blobs is predicted for each frame, and the update rate *α* of the segmentation algorithm is significantly lowered for these areas. Consequently, objects must stay for a very long time before merging into the background.

To predict blob positions for the current frame, *t*, blobs from *t* and t−1 are matched. Subsequently, the displacements between *t* and t−1 are applied on *t*. The matching is done with a nearest neighbor search of the blob’s centroids. If no neighbor within range *ρ* is found, the blob is supposed to be stationary as no prediction about the movement can be made.

We extend the method by Yao and Ling [29] by dilating the predicted blobs and smoothing out edges. This is done to prevent artifacts in the background model caused by inaccuracies in the blob prediction. The update rate *α* of the segmentation algorithm is thus calculated as:

(22) $$\alpha =D_{\mathrm{predict}}\alpha_{\mathrm{fg}}+\left(1-D_{\mathrm{predict}}\right)\alpha_{\mathrm{bg}}$$

where $0\leq D_{\mathrm{predict}}\leq 1$ indicates the value in the blob prediction image and $\alpha_{\mathrm{fg}}$ and $\alpha_{\mathrm{bg}}$ are the update rates for foreground and background regions, respectively.

**Figure 10:**
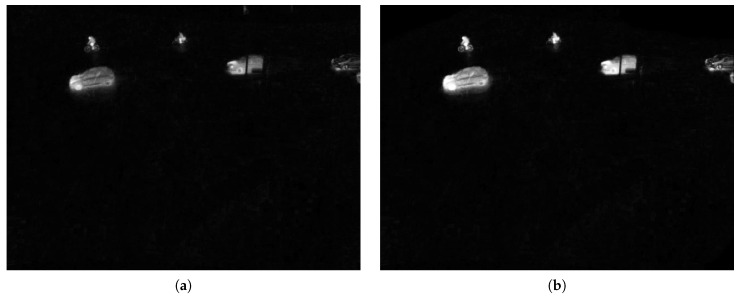
Distance map before (**a**) and after blob prediction-based modulation (**b**).

Another purpose of the blob prediction is presented in this work. As the boundary of foreground objects changes only gradually, the predicted blobs provide a very good estimate of the foreground of the next frame. This can help the segmentation, as it is more likely to locate an object where predicted than elsewhere in the scene. Objects follow a trajectory and generally do not appear unexpectedly. To express this characteristic, another modification of the distance map is performed. Analogous to the shadow suppression, the predicted areas are up-scaled in the distance map. Figure 10 demonstrates the effect. The right image of Figure 10 shows the distance map after the blob prediction. Compared to the distance map before the prediction, as shown on the left of Figure 10, one sees that objects appear brighter in the right image and thus have a higher likelihood of being declared foreground.

### 6.3. Scene Geometry-Based Knowledge

**Figure 11:**
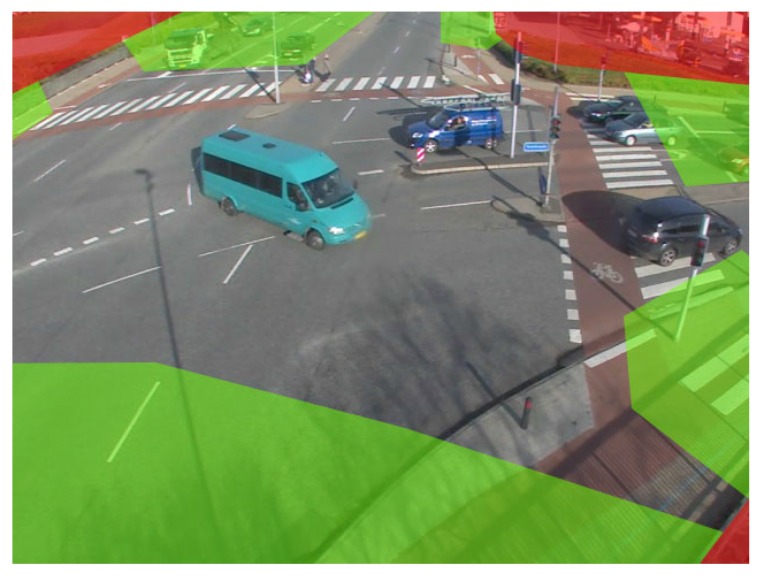
Scene area classes. Green: entrance areas; red: excluded areas; rest: neutral.

The principle presented in the last sections can be used for another constraint. By looking at the scene geometry, one can easily divide the image into three classes. The first class of pixels is areas where no foreground is expected under any circumstances, for example trees or the sky. The second class of pixels denotes the areas into which objects may move. A sudden appearance of objects is unlikely or even excluded, but objects may move to these areas from other parts of the image. These areas are referred to as neutral zones. The last class describes the areas in which we expect foreground objects to appear. These areas are called entrance areas in the following. Entrance areas can normally be found at the borders of the image as objects enter the scene, normally from outside the viewport of the camera. Objects may, however, also reappear after occlusion or enter from occluded areas. Based on this classification, a mask can be drawn as seen in Figure 11.

Firstly, excluded areas cannot be categorized as foreground when the corresponding values in the distance map are set at zero. Secondly, the distance values for neutral zones are scaled down by $s_{\mathrm{neutral}}$ to make it less likely to find foreground pixels in these areas. This is possible because the blob positions have been predicted and uprated beforehand. Areas to which we expect objects to move are untouched afterwards or even uprated, whereas unpredicted regions are down-rated. This helps remove noise, and found objects are considered more reliable.

## 7. Experiments

A series of experiments has been conducted to evaluate both the quantitative and the qualitative performance of the proposed algorithm. This section begins with an elaboration about the datasets that have been used in this work, followed by a description of the performance metrics and the results of the experiments. Finally, an in-depth analysis of the qualitative performance is presented.

### 7.1. The Datasets

The main dataset used in this work contains a large number of thermal-RGB recordings of intersections in Northern Jutland, Denmark, recorded during 2013. The videos are undistorted using the line-based parameter estimation by Alemán-Flores et al. [30]. To be able to benchmark the proposed algorithm, we include two commonly-used datasets. The Ohio State University (OSU) Color-Thermal Database [31] of the Object Tracking and Classification Beyond the Visible Spectrum (OTCBVS) Benchmark Dataset Collection contains RGB and thermal data of two surveillance scenarios. The videos contain pedestrians recorded on the campus of Ohio State University. The National Optics Institute (INO) Video Analytics Dataset (http://www.ino.ca/en/video-analytics-dataset/) contains a set of multimodal recordings of parking lot situations, including data on cars, cyclists and pedestrians.

As we know the exact location and time of our own datasets, we can compute the altitude of the Sun directly and retrieve weather information from a nearby weather station. As this contextual information is not known for the external datasets, we derive the contextual information from manual scene observations. Weather conditions are grouped into the categories introduced in Table 1. All scenes tested during the experiments are listed in Table 2 and Table 3. The contextual information for each scene is listed in Table 4.

### 7.2. Performance Metrics

We evaluate the experiments by the quantitative performance metrics used in [32]. These metrics are the Detection Rate (DR) and the False Alarm Rate (FAR), defined as:

(23) $$\mathrm{DR}=\frac{\mathrm{TP}}{\mathrm{TP}+\mathrm{FN}}$$

(24) $$\mathrm{FAR}=\frac{\mathrm{FP}}{\mathrm{TP}+\mathrm{FP}}$$

with True Positives (TP), False Positives (FP) and False Negatives (FN). The DR is also known as recall or the true positive rate and describes the sensitivity of a detector. The FAR corresponds to 1−p, where *p* is the detector’s precision or specificity.

In order to evaluate the performance metrics, access to the true data, commonly referred as the Ground Truth (GT), is needed. GT must be created manually and is a laborious task. Thus, only a small sample of the results can be tested. In this work, 70 successive frames have been annotated for each test set with the exception of 180 annotated frames of the Auto Gain set. In our own dataset, this amounts to approximately 3 and 7 s of video, respectively. The GT has been annotated using the Aalborg University Visual Analysis of People (AAU VAP) Pixel Annotator (https://bitbucket.org/aauvap/multimodal-pixel-annotator) where the boundary of each object has been traced manually by a mouse. The average number of objects per frame is shown for each sequence in Table 4.

### 7.3. Quantitative Results

In order to evaluate the performance of the proposed method, extensive experiments have been performed and evaluated with the described performance metrics. Besides the algorithm itself, each dataset has been processed by applying four alternative strategies, presented below:

- RGB: individual processing of the RGB modality by the proposed method.
- Thermal: individual processing of the thermal modality by the proposed method.
- RGBT: pixel-wise, naive (not context-aware) fusion of RGB and thermal streams.
- Select: confidence-based selection as presented by Serrano-Cuerda et al. [11]

All strategies are based on the GMM background segmentation algorithm presented by Stauffer and Grimson [28] and improved by Zivkovic [33] and differ only in the ways the data fusion is performed. This allows us to measure the contribution of our context-aware fusion approach compared to other fusion approaches or single-modality processing. As mentioned in Section 5, other segmentation algorithms may be used in combination with the proposed method. By using a well-known approach, such as the GMM, however, we believe that the comparison reveals interesting insights on the strengths and weaknesses of the proposed method.

This work aspires to create a system that works without requiring the manual tuning of its parameters for different conditions. Therefore, only the learning time for each scene has been adjusted to match the specific situation. For example, scenes with much traffic need more time to learn a stable background model. For the case of the presented algorithm, background models have been learned individually before the described adjustments were made. This procedure is necessary because the predicted foreground regions are learned slowly, and false positives are very likely to appear during the learning phase.

The update rate *α* of the segmentation algorithm has been set to be slower for the alternative strategies. As the GMM background modeling does not differ between foreground and background in the update step, a quick update rate would result in foreground objects merging into the background. This is also the case in the learning phase of the proposed method. Consequently, the same *α* has been used here. All important experimental parameters are listed in Table 5, where the parameters below the line apply only for the proposed method.

Shadow detection has been performed for all experiments containing RGB data. Pixels that have been categorized as shadow have been classified as background in the reference methods. Furthermore, the scene area classes have been applied on the resulting data. This approach ensures that equal conditions have been created for all strategies, and identified differences in the results of the proposed algorithm in contrast to the alternative strategies can be explained by its core contributions.

The results of the experiments are displayed in Table 6. The general performance of the proposed algorithm can be considered very good due to a average DR and FAR of 0.95 and 0.35, respectively. The table clearly shows that the goal of creating a robust method for a wide bandwidth of conditions has been achieved. Only the proposed method shows good performance for every test sequence, which is expressed in the average FAR and DR rates, which are significantly better than the alternative strategies. These strategies fail in different scenarios, but show better performance than the proposed method for some scenarios. The reasons for this are manifold and will be discussed in Section 7.4.

As expected, all fusion approaches generally tend to demonstrate better performance than single-modality methods. The method presented by Serrano-Cuerda et al. [11] also seems to perform well at first glance. However, when analyzing the results in detail, it becomes clear that the results are, at best, as good as one of the single modalities. This is related to the design of the algorithm; it is designed to select one result of two parallel pipelines. One important characteristic of fusion algorithms is neglected by this design choice. Fused data or fused results generally differ from the original inputs and may, therefore, contain new features and novel information. A simple selection obviously makes this impossible.

The FAR of both the proposed method and the RGBT approach mirror the weaker modality. The reason is that high evidence of foreground objects in one modality may still be present after fusion of the data. Only false positives based on weak evidence are successfully smoothed out. In the worst case, false positives from both modalities are present in the result.

A good example of the superiority of fusion approaches in terms of DR is given by the sequence INO TreesAndRunner. Obviously, both fusion approaches, i.e., the proposed method and the RGBT approach, perform much better than the single modalities. This better performance is seen because both RGB and thermal contain frames that are very hard to segment. The runner, for example, will pass trees and other objects. Nevertheless, the fusion approaches can still rely on the second modality when the information content of the first is low.

### 7.4. Special Situation Performance

In the following, the results of the specific test sequences are elaborated in detail. It is shown how the performance of the proposed algorithm is affected by different details of the design. Four different problems that tend to arise during outdoor surveillance are discussed. Emphasis has been put on the adaptive modality weighting of the proposed algorithm and its effect on the segmentation results. This context awareness is initially discussed below.

#### 7.4.1. Context Awareness

One of the core contributions of this work is the context awareness of the fusion. It is based on a set of quality indicators that have been defined in Section 3. The goal is to evaluate the usefulness of each modality. Instead of using information from the images themselves, contextual information from outside sources has been consulted. Solely the thermal domain has been rated by its own information content. For the tested sequences, the weights calculated by the indicators are more or less fixed. The time frames are simply too short to see an effect based on, for example, the quality indicator covering the altitude of the Sun. The overall concept, however, has been tested by selecting scenes with various conditions.

**Figure 12:**
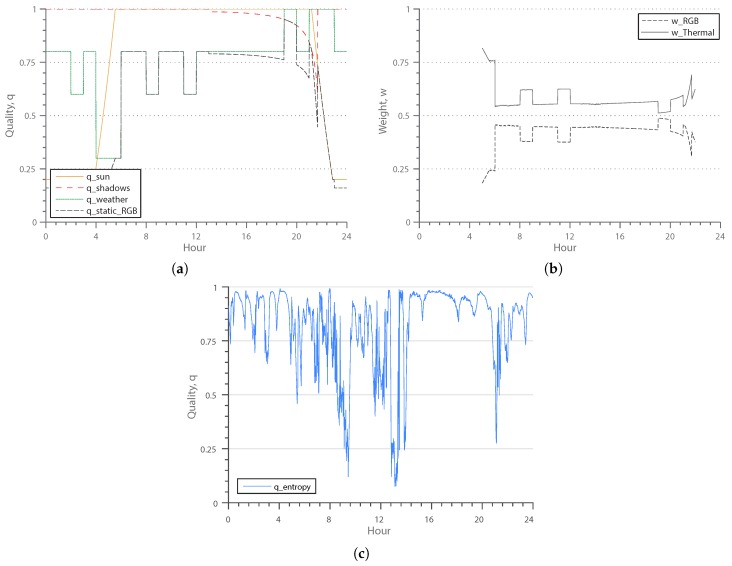
RGB and thermal quality indicators and resulting weights of a full day. (**a**,**b**) have been computed on the same sequence, whereas (**c**) shows the thermal quality indicator for the day that includes the “snowing” sequence. The snowfall starts at 13:17 and severely effects the quality of the thermal image. (**a**) Predictable RGB quality indicators and resulting RGB quality, $q_{{\mathrm{static}}_{\mathrm{RGB}}}$, over a full day; (**b**) weights of the RGB and thermal modalities, $w_{\mathrm{RGB}}$ and $w_{\mathrm{Thermal}}$, over a full day; (**c**) entropy-based quality indicator, $q_{\mathrm{entropy}}$, for the thermal domain.

In Figure 12a,b, quality functions covering a full day are plotted. The plotted day was a summer day with rather good weather. Because of an overcast sky, no cast shadows were detected for this particular morning. Around noon time, the temperature was so high that the thermal camera was overexposed. Figure 12c shows the entropy-based quality indicator for a full day, which includes the ’snowing’ sequence. The sequence starts at 13:17, and one may identify sharp drops in the quality indicators related to the snowfall.

#### 7.4.2. Parameter Sensitivity

The experimental results have been obtained using the quality indicators listed in Table 4. In the following, we will perform a sensitivity analysis to judge the effect of changing the parameters that guide the context-aware fusion. We use the Snowing and INO ParkingEvening scenes and vary the parameters $q_{\mathrm{sun}}$ and $q_{\mathrm{weather}}$ in the interval [0.1,1.0]. When both $q_{\mathrm{sun}}$ and $q_{\mathrm{weather}}$ are low, the resulting weight of the RGB image, $w_{\mathrm{RGB}}$, will be low and the distance map of the thermal image will dominate the fusion process. When both indicators are set to one, the resulting weights will depend on the thermal entropy and the unpredictable quality indicators for both modalities. However, the resulting value of $w_{\mathrm{RGB}}$ will be relatively higher.

**Figure 13:**
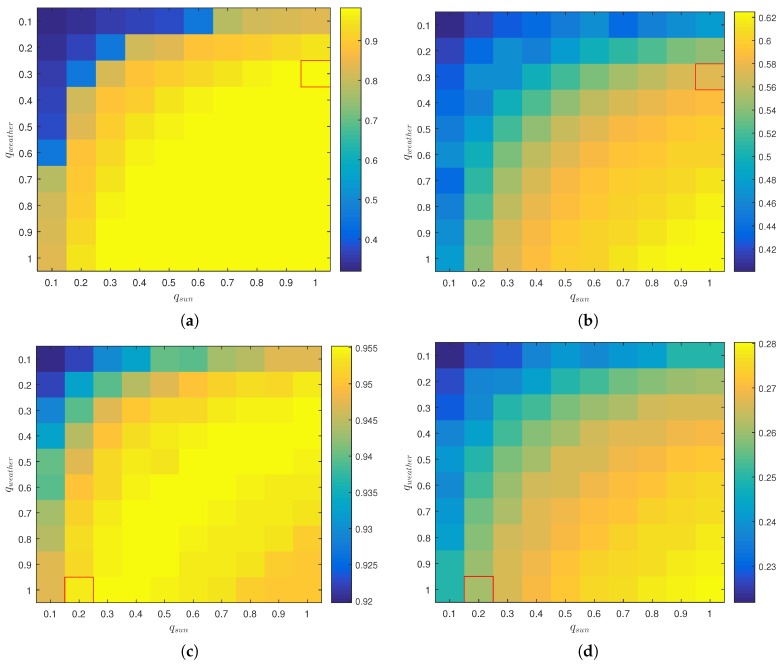
Experimental results on the Snowing and INO ParkingEvening sequences. DR and FAR are shown with varying values of the quality indicators $q_{\mathrm{sun}}$ and $q_{\mathrm{weather}}$. (**a**) DR, Snowing; (**b**) FAR, Snowing; (**c**) DR, INO ParkingEvening; (**d**) FAR, INO ParkingEvening.

The DR and FAR rates of the two scenes for varying values of $q_{\mathrm{sun}}$ and $q_{\mathrm{weather}}$ are shown in Figure 13. The values used by the general experiments listed in Table 6 are enclosed by a rectangle.

The figures reveal differences on the reliance on the RGB and thermal modalities for the two scenes. The Snowing scene is more reliant on the RGB image than INO ParkingEvening, which shows comparatively little improvement in DR rates when integrating the RGB image into the fusion. By setting $q_{\mathrm{sun}}=q_{\mathrm{weather}}=0.1$, the Snowing sequence returns a DR below 0.4, whereas the INO ParkingEvening sequence holds a relatively high DR of 0.92. In general, the results show the importance of integrating context-aware quality indicators; a naive fusion, as exemplified by $q_{\mathrm{sun}}=q_{\mathrm{weather}}=1$, does not always give the best compromise of DR and FAR.

#### 7.4.3. Automatic Gain Control

**Figure 14:**
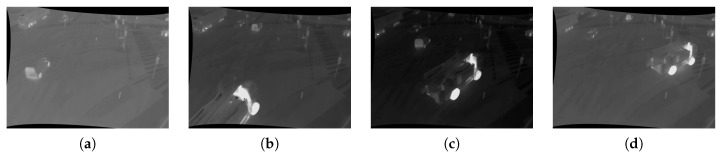
Automatic Gain Control (AGC) of the IR camera triggered by a big truck coming into the scenery. (**a**) Frame 170; (**b**) Frame 200; (**c**) Frame 230; (**d**) Frame 260.

When large or hot objects enter the scenery, the camera automatically adjusts its gain in order to preserve a high level of detail. This behavior, however, seriously disturbs the segmentation algorithm, which results in a high number of false positive foreground pixels. Figure 14 displays the described phenomena. The main challenge is thus the short time frame for adjustment. When the objects leave the scene, the camera re-adjusts the gain. Therefore, the problem often persists only for 100–200 frames, and yet, it highly affects the segmentation results.

**Figure 15:**
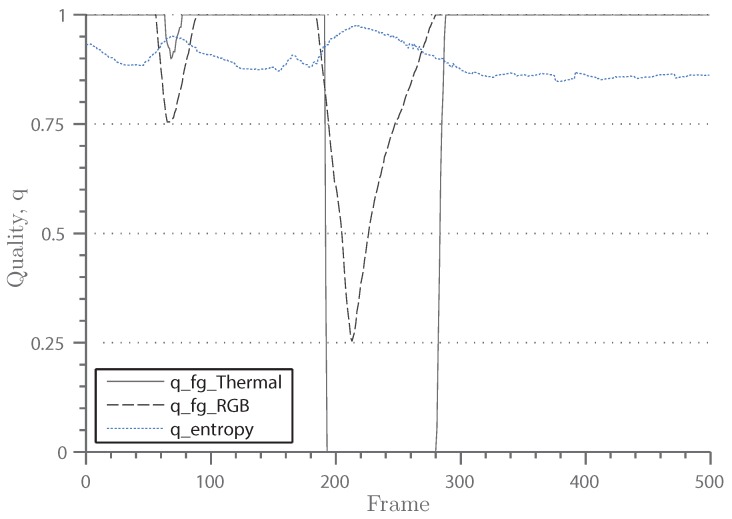
The entropy quality indicator, $q_{\mathrm{entropy}}$, and the quality indicators of the unpredictable conditions, $q_{{\mathrm{fg}}_{\mathrm{RGB}}}$ and $q_{{\mathrm{fg}}_{\mathrm{Thermal}}}$, for the Auto Gain test sequence. The $q_{{\mathrm{fg}}_{\mathrm{Thermal}}}$ drops rapidly when a truck enters the scene around Frame 230. The corresponding frames are seen from Figure 14.

As seen in Table 6, the proposed algorithm handles the described problem well. No segmentation quality reduction can be detected from the raw numbers. The reason for this is the adaptive weighting performed in the fusion step. Through the foreground ratio evaluation, which was described in Section 3.3, it can be detected that the background model of the thermal domain is invalid. As a result, the thermal weight function drops to zero, and the segmentation relies on the RGB domain only. Figure 15 displays this behavior. It can clearly be seen that the quality function drops parallel to the weight of the thermal domain. When the truck has left the scene, the camera adjusts back to normal, and the quality function instantly rises. The weight, however, increases only gradually. This delay is necessary in order to give the background model sufficient time to relearn the background model.

#### 7.4.4. Changing Illumination

A very similar problem, which is commonly seen in outdoor surveillance, is changing illumination conditions. The segmentation algorithm is designed to adapt only to slow changes, e.g., shadows moving during daytime, whereas fast changes in the scenery will cause false detection of foreground objects.

Similar to the problem of automatic gain control, which was discussed above, the foreground ratio of the RGB domain will rise because the background model does not adapt fast enough for these changes. Consequently, a weight shift to the thermal domain will be performed by the algorithm, which contributes to the comparatively low FAR of 0.56 in the OTCBVS 3 sequence.

#### 7.4.5. Artifact Reduction

**Figure 16:**
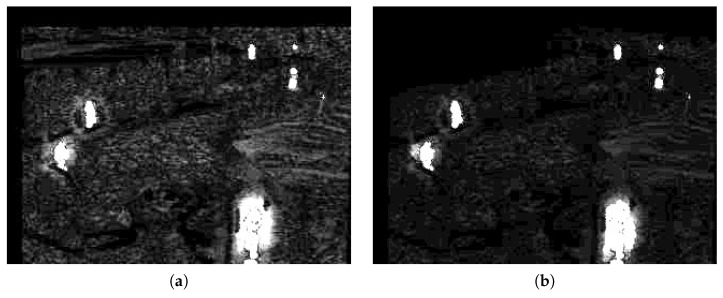
Distance map before (**a**) and after scene geometry-based modulation (**b**).

Another contribution of the proposed algorithm can be seen in the results of OTCBVS 3. With 0.56, the FAR is even lower than the results found for the thermal background subtraction. This can be reasoned with the adjustments made to the fused distance map on the basis of the scene geometry. Artifacts are unlikely to appear since unpredicted foreground regions are reduced in the distance map. The effect on the foreground mask can clearly be seen in Figure 16, particularly when compared to the approach using solely the thermal domain.

#### 7.4.6. Long-Staying Objects

**Figure 17:**
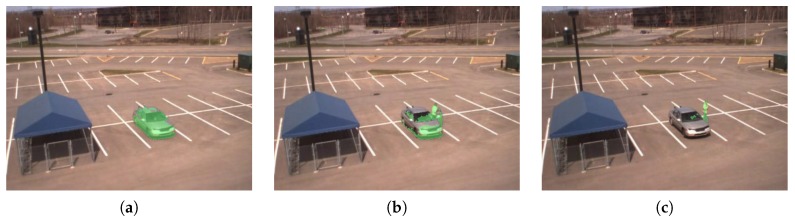
The parked car gradually merges into the background by using a standard GMM segmentation method (foreground marked green). (**a**) Car arrives; (**b**) Car has stopped for several frames; (**c**) Driver got out the car. The proposed method mitigates this issue by predicting foreground regions and lowers the update rate *α* correspondingly.

The GMM background subtraction presented by Stauffer and Grimson [28] assumes that foreground objects are constantly in motion, but this is obviously not the case for all traffic. This issue has been addressed by Yao and Ling [29], and the proposed method has been integrated in our work. The original algorithm causes long-staying objects to gradually merge into the background. This problem is very visible in the INO CoatDeposit test set. The car entering the scene merges into the background within a few frames as seen in Figure 17. This merging is stopped by prediction of foreground regions and lowering of their update speed, resulting in a significantly better DR of the proposed method.

## 8. Conclusions and Future Perspectives

This paper presented a new approach to multi-modal image fusion. The proposed algorithm fuses the soft segmentation results of two parallel segmentation pipelines based on the RGB and thermal video streams. The fusion is guided by quality indicators for each modality. The quality indicators are based on both image structure and external sources of information. These include the entropy of the thermal image, the altitude of the Sun, the weather conditions of the scene and rapid changes in the resulting output of the parallel segmentation pipelines. To match the requirements derived from the purpose of traffic monitoring, extensions to the core contribution have been introduced.

The proposed method has been thoroughly tested. The results show that the proposed method performs significantly better than naive fusion of both modalities and consistently better than utilizing a single modality alone. The evaluated performance suggests that the strategy of including image quality indicators in the segmentation process has great potential in future applications.

A common problem of image fusion techniques can be seen from the experimental results. Although the algorithm features a suppression of false positives, a propagation to the fused mask can still be noticed. This is especially the case when the quality rating of the two modalities is similar and information therefore fuses in equal proportions. Based on this observation, further development of the proposed method can be derived. Serrano-Cuerda et al. [11] perform a switch based on image quality indicators, whereas this work performs an adaptive fusion. The next logical step would be to perform the fusion adaptive per image region. By specifying quality indicators for image samples, information about shadows and different lighting conditions within the scene could be considered.

The work has been limited to the usage of RGB and thermal imagery. However, the algorithm can easily be adapted to work with different imaging sensors. A setup of the proposed system in combination with sensors helping to estimate the image quality would also be an interesting extension. Weather stations and street temperature sensors would enable the indicators to work much more accurately.

## Figures and Tables

**Table 1:** Weather conditions and their corresponding category and quality indicator, $q_{\mathrm{weather}}$.

Weather Condition [25]	Category	$q_{\mathrm{weather}}$
Clear	Good conditions	1.0
Overcast	Low/varying illumination	0.8
Cloudy		
Light mist, drizzle		
Heavy drizzle, mist	Reflections/moisture	0.6
Light rain		
Snow	Particle occlusion/precipitation	0.3
Hail		
Heavy rain		
Thunderstorm		
Fog, haze	Reduced visibility	0.3
Dust, sand, smoke		

**Table 2:** Test scenes from our own dataset. The videos are rectified using a line-based parameter estimation method [30].

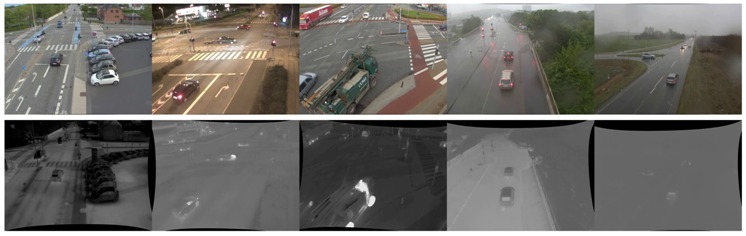				
Day	Night	Auto Gain	Heavy Rain	Snowing

**Table 3:** Test scenes from the benchmark datasets.

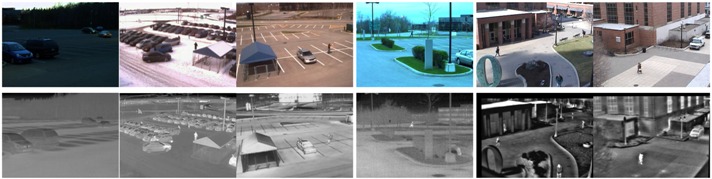					
INO ParkingEvening	INO ParkingSnow	INO CoatDeposit	INO TreesAndRunner	OTCBVS 3	OTCBVS 4

**Table 4:** Annotation properties and corresponding context-based quality characteristics for each test scene. For our own dataset, Sun altitude and weather information are provided through direct computations and a weather database, respectively. For the benchmark dataset, this information has been derived from manual scene observations.

Sequence	Annotated Frames	Average Number of Objects per Frame	Weather Classification	$q_{\mathrm{weather}}$	Sun Altitude	$q_{\mathrm{sun}}$	$q_{\mathrm{shadows}}$
Day	70	6.4	Good conditions	1.0	$20^{\circ }$	1.0	0.95
Night	70	6.9	Low illumination	0.8	$-19^{\circ }$	0.20	1.0
Auto Gain	180	9.0	Moisture	0.6	$20^{\circ }$	1.0	1.0
Heavy Rain	70	6.7	Moisture	0.6	$29^{\circ }$	1.0	1.0
Snowing	70	5.6	Precipitation	0.3	$9^{\circ }$	1.0	1.0
INO ParkingEvening	70	2.1	Good conditions	1.0	$-12^{\circ }$	0.20	1.0
INO ParkingSnow	70	7.0	Low illumination	0.8	$86^{\circ }$	1.0	1.0
INO CoatDeposit	70	2.8	Low illumination	0.8	$46^{\circ }$	1.0	0.98
INO TreesAndRunner	70	1.0	Low illumination	0.8	$0^{\circ }$	0.50	0.30
OTCBVS 3	70	3.9	Low illumination	0.8	$12^{\circ }$	1.0	0.91
OTCBVS 4	70	1.0	Good conditions	1.0	$12^{\circ }$	1.0	1.0

**Table 5:** Parameters used in the experiments. The parameters below the line apply only for the proposed method.

Parameter	Value	Description
*α*	0.0005	GMM update rate
*K*	5	Number of components for GMM
*λ*	4	Number of standard deviations for background acceptance for GMM
*T*	1	Segmentation threshold of the distance map
$\alpha_{\mathrm{BG}}$	0.0033	Background update rate for blob-based prediction
$\alpha_{\mathrm{FG}}$	0.000033	Foreground update rate for blob-based prediction
*τ*	0.1	Foreground ratio
*γ*	5.0	Foreground deviation weight
*ρ*	17	Blob match radius (px)
$s_{\mathrm{shadow}}$	0.3	Distance scaling factor for shadow regions
$s_{\mathrm{predict}}$	1.5	Distance scaling factor for predicted regions
$s_{\mathrm{neutral}}$	0.5	Distance scaling factor for neutral regions
$q_{\mathrm{smin}}$	0.2	Minimum quality of $q_{\mathrm{sun}}$
$q_{\mathrm{shmin}}$	0.3	Minimum quality of $q_{\mathrm{shadow}}$

**Table 6:** Experimental results; first line, Detection Rate (DR), and the second line, False Alarm Rate (FAR). The best DR and FAR values of each set are marked in bold. The proposed method is compared to individual processing of RGB and thermal (RGBT) frames, naive fusion of RGBT frames and “select”, which indicates result selection based on quality heuristics [11].

	Proposed	RGB	Thermal	RGBT	Select
Day	**0.99**	0.93	0.95	0.97	0.93
	0.30	**0.09**	0.31	0.29	**0.09**
Night	0.84	0.78	0.48	**0.89**	0.78
	**0.31**	0.69	0.32	0.66	0.69
Auto Gain	**0.94**	0.86	0.73	0.91	0.81
	0.25	**0.09**	0.76	0.40	0.58
Heavy Rain	**0.92**	0.46	0.69	0.48	0.69
	0.22	0.26	**0.11**	0.27	**0.11**
Snowing	**0.96**	0.79	0.21	0.92	0.21
	0.52	0.52	**0.25**	0.55	**0.25**
INO ParkingEvening	**0.95**	0.93	0.91	**0.95**	0.91
	0.26	0.27	**0.18**	0.29	0.18
INO ParkingSnow	0.98	0.86	**0.99**	0.96	**0.99**
	**0.32**	0.78	0.40	0.35	0.40
INO CoatDeposit	**0.97**	0.10	0.10	0.10	0.10
	0.19	**0.12**	0.30	0.16	**0.12**
INO TreesAndRunner	**0.94**	0.88	0.84	0.93	0.84
	0.44	0.65	**0.36**	0.70	**0.36**
OTCBVS 3	**0.95**	0.75	0.94	0.90	0.78
	**0.56**	0.96	0.74	0.96	0.93
OTCBVS 4	**1.00**	0.94	0.78	0.99	0.78
	0.55	**0.15**	0.68	0.48	0.68
Average	**0.95**	0.76	0.70	0.83	0.72
	**0.35**	0.39	0.39	0.46	0.41
